# Topography and Expansion Patterns at the Biofilm-Agar Interface in *Bacillus subtilis* Biofilms

**DOI:** 10.3390/microorganisms9010084

**Published:** 2020-12-31

**Authors:** Sarah Gingichashvili, Osnat Feuerstein, Doron Steinberg

**Affiliations:** 1 Biofilm Research Laboratory, Faculty of Dental Medicine, Institute of Dental Sciences, Hebrew University-Hadassah, Jerusalem 9112001, Israel; dorons@ekmd.huji.ac.il; 2 Department of Prosthodontics, Faculty of Dental Medicine, Hebrew University-Hadassah, Jerusalem 9112001, Israel; osnat.feuerstein@mail.huji.ac.il

**Keywords:** biofilm, topography, *Bacillus subtilis*, surface attachment

## Abstract

Bacterial biofilms are complex microbial communities which are formed on various natural and synthetic surfaces. In contrast to bacteria in their planktonic form, biofilms are characterized by their relatively low susceptibility to anti-microbial treatments, in part due to limited diffusion throughout the biofilm and the complex distribution of bacterial cells within. The virulence of biofilms is therefore a combination of structural properties and patterns of adhesion that anchor them to their host surface. In this paper, we analyze the topographical properties of *Bacillus subtilis’* biofilm-agar interface across different growth conditions. *B. subtilis* colonies were grown to maturity on biofilm-promoting agar-based media (LBGM), under standard and stress-inducing growth conditions. The biofilm-agar interface of the colony type biofilms was modeled using confocal microscopy and computational analysis. Profilometry data was obtained from the macrocolonies and used for the analysis of surface topography as it relates to the adhesion modes present at the biofilm-agar interface. Fluorescent microspheres were utilized to monitor the expansion patterns present at the interface between the macrocolonies and the solid growth medium. Contact surface analysis reveals topographical changes that could have a direct effect on the adhesion strength of the biofilm to its host surface, thus affecting its potential susceptibility to anti-microbial agents. The topographical characteristics of the biofilm-agar interface partially define the macrocolony structure and may have significant effects on bacterial survival and virulence.

## 1. Introduction

Bacterial biofilms are found in abundance in nature—these microbial communities, which are often characterized by a complex, self-secreted extracellular matrix, readily form on various natural and synthetic surfaces. Biofilms’ relatively low susceptibility to anti-microbial treatments makes them a major cause for concern in many fields including the food industry—due to contamination [1], healthcare—due to infections [2] and maritime transport—due to their formations on ship hulls [3].

The matrix in which bacterial cells are embedded consists of exopolysaccharides and other types of biomolecules such as lipids, proteins and nucleic acids. The interaction between components of the extracellular matrix was shown to play a role in the three-dimensional structuring of biofilms [4,5]. In *B. subtilis* biofilms, presence of specific biomaterials such as calcite minerals [6] and amyloid fibers [7] was shown to provide structural integrity and “robustness”.

Bacteria in the biofilm microenvironment, as opposed to their planktonic state, benefit from increased resilience—not only due to limited diffusion of anti-microbial agents, but also due to the complex bacterial organization within the biofilms. For example, *B. subtilis* biofilms mature by way of cellular differentiation, which allows a number of cell-types to simultaneously coexist within the biofilm [8]. A complex organization of matrix-producing, motile and sporulating cells in the colony as well as communication through quorum-sensing mechanisms [9] allows biofilms to adapt to their environment not only at the individual cellular level but also on a macro, whole colony scale by altering colony phenotype—namely, colony morphology and surface topography. Indeed, the strong and multi-faceted phenotypical diversity of *B. subtilis* colony type biofilms is considered to be key to understanding the “robustness” of such biofilms.

Modern biofilm research focuses on finding efficient treatments for the partial or complete eradication of potentially harmful biofilms as well as prevention of their formation. The ability to accurately characterize and quantify biofilms, is therefore paramount and traditionally done by analyzing their biomass. To that end, biofilms are removed from their host surfaces and subjected to various disruptive procedures such as sonication, vortexing and swabbing. However, such methods, by their very nature, disregard two important aspects of biofilm formation—the undisrupted whole colony morphology and the strength of adhesion to the growth substrate.

Morphological changes that occur in response to changing environmental conditions have been previously observed in *B. subtilis* colony type biofilms. For example, Wang et al. [10] reported changes in biofilm thickness, rim organization and distribution of cell phenotypes within a mature macrocolony occurring at different substrate stiffness and nutrient levels. Similarly, changes in colony growth dynamics such as biofilm expansion rate and peak expansion velocity were also observed [11]. Specific morphological changes such as the appearance of wrinkles that connect colony core to periphery under limited nutrient supply were described by Gingichashvili et al. [12]. Surface topography in *B. subtilis* colony type biofilms was also shown to be dependent on environmental conditions and determinant of several key properties such as biofilm wettability [13] as well as erosion sensitivity and antibiotic efficiency [14]. The presence of metal ions in the growth medium was shown to significantly affect colony hydrophobicity [15] at the biofilm-air interface, which showed a gradual topographical roughening over time [16]. These studies in biofilm morphology and surface topography, as well as many others, have demonstrated the importance of understanding the correspondence of certain growth conditions to the resulting colony phenotype.

When it comes to assessing the strength of adhesion between colony type biofilms and their growth substrates, methods are scarce. Several examples of biofilm adhesion or erosion assays were reported in the literature—one example is a method by Klotz et al. [17] who use rotational motion of a lab shaker to induce shear forces onto biofilm agar patches and measure the resulting biofilm displacement as an indicator for erosion. Alternatively, Falcon Garcia et al. [14] use a water reservoir with tubing system to erode biofilms by controlled dripping or continuous flow. While such erosion assays have the benefit of being applied to undisrupted biofilms, methods to quantify biofilm displacement as a result of physical erosion forces suffer from challenges in obtaining accurate results—for example, macrocolonies are often torn and fold onto themselves, making it difficult to rely on eroded area measurements. Disruptive methods to assess adhesion strength are more commonly found—Cerca et al. [18] use dry-weight measurements to determine the adhered cell mass. Other methods include a flow cytometric assay by Silva-Dias et al. [19] for the quantification of yeast cells that attach to polystyrene microspheres and a DNA-based method by Vanysacker et al. [20] for the quantification of *P. aeruginosa* and *E. coli* biofouling. More complex methods include quartz crystal microbalance with dissipation monitoring [21], surface plasmon resonance [22] and atomic force microscopy [23].

In this paper, we focus on biofilm surface topography directly at the interface between the macrocolony and agar substrate. This allows us to visualize specific topographical characteristics of the biofilm-agar interface itself, unseen when observing macrocolonies from the biofilm-air interface. Such direct analysis of the adhering surface also provides a possible link between topography and adhesion.

## 2. Materials and Methods

### 2.1. Strain and Growth Media

Starter cultures of *B. subtilis* YC161 (*P_spank_*-gfp) [24] were grown in Lysogeny broth (LB; 10 g of tryptone (Neogen, Lansing, MI, USA), 5 g of yeast extract (Neogen, Lansing, MI, USA) and 5 g of NaCl per liter and incubated at 37 °C at 150 rpm for five hours. The LB growth medium was solidified by the addition of 1.5% (*w*/*v*) agar and further supplemented with 1% (*v*/*v*) glycerol and 0.1 mM MnSO_4_ [25]. Nutritionally depleted colony type biofilms were grown on media with reduced LB content in the solvent—“fully nutritional” colonies were grown with 100% nutrient supply (8 g LB per 400 mL solvent) while their nutrient depleted counterparts were seeded on media containing 4 g (50% nutrient supply) and 2 g (25% nutrient supply) LB in the same amount of solvent.

### 2.2. Biofilm Formation

An amount of 2.5 µL of suspension from the starter culture (O.D. 600 nm = 1) was seeded onto biofilm-promoting agar plates and incubated at 30 °C for a period of three days.

### 2.3. 2D Fluorescent Microscopy

Images of colony type biofilms were taken using Nikon SMZ25 microscope with ORCA-R2 camera (Nikon Corporation, Tokyo, Japan). GFP fluorescence was measured using 470 nm excitation and 525 nm emission. Cy5 fluorescence was measured using a 620 nm excitation wavelength and 665 nm emission. 0.5× magnification was used, 9.46 µm/px in *X*/*Y* axes. Channel connectivity at the biofilm-agar interface was visualized in Imaris’ *Image Processing* module using the following three-step image processing pipeline on the obtained 2D images: (a) channel inversion (b) background subtraction and (c) linear stretch.

### 2.4. Optical Profilometry

Upon reaching morphological maturity, *B. subtilis* colonies were removed from the agar plates. Their footprint upon the solid agar medium was then visualized under optical profilometer (Optical Profilometer Contour GT-K1, Bruker Corporation, Billerica, MA, USA) and analyzed using Vision64 Map software (Bruker Corporation, Billerica, MA, USA).

### 2.5. Confocal Microscopy

Bacterial biofilms were removed gently from agar surfaces to glass cover slips using phosphate-buffered saline (PBS tablets, Merck KGaA, Darmstadt, Germany). All images were taken using a Nikon Spinning Disk Confocal microscope (Yokogawa W1 Spinning Disk, Nikon Corporation, Tokyo, Japan). GFP fluorescence was measured using 488 nm excitation and 525 nm emission. Cy5 fluorescence was measured using a 638 nm excitation wavelength. 10x magnification was used, 1.30 µm/px in *X*/*Y* axes, 2.5 µm between adjacent confocal slices (*Z*-axis).

### 2.6. 3D Modeling and Computational Analysis

Three-dimensional views of colony type biofilms were constructed using Imaris software (Imaris 9.6.0, Oxford Instruments, Abingdon, UK, 2020) from confocal images. The *Contour Surface* module in Imaris was used to create a three-dimensional rendering of channels’ interior. This was done by first manually drawing contours over channel interiors in two-dimensional slices of the confocal images. Contours were drawn sequentially on slices every 12.5 µm and subsequently used for Imaris’ three-dimensional interpolation that yields a volumetric rendering of channel interior. It is important to note that multiple contours were drawn in each two-dimensional slice to represent the interior of each individual channel that appears in the image.

The positions of centroids of fluorescent microspheres were detected using Imaris’ *Spots* module. Microspheres that were co-localized inside the volumetric rendering of channel interior (constructed as described above) were labeled in blue, while microspheres whose centroids fell outside of the channel interior rendering were labeled in yellow.

### 2.7. Fluorescent Microspheres

Fluorescent carboxylate-modified red microspheres (660/690), 1 µm in diameter (Bangs Laboratories, Inc., Fishers, IN, USA), were prepared in a PBS buffer 10% (*v*/*v*). Colony type biofilms were incubated with 100 µL/3 mL microspheres for a mixture of bacteria and microspheres. Microsphere droplets (2.5 µL) were seeded on the solid medium for droplet deformation analysis—2D images of the maturing colonies were taken as described in Section 2.3.

### 2.8. Statistical Analysis

Statistical analyses were performed using Microsoft Excel software using Student’s *t*-Test. Statistically significant values are determined by a *p*-value of less than 0.05.

## 3. Results

### 3.1. Visualizing the Contact Surface

When visualizing the biofilm-agar interface, the organizational patterns by which the biofilm is being structured during maturation become apparent. In this paper, we investigated changes that may take place at the center of macrocolonies’ ’core’—a central region of mature colony type biofilms that is morphologically uniform but distinct from colony periphery. The two images on the left show the center of the macrocolony (red) at 100% (top) and 50% (bottom) nutrient supply. In order to better visualize the microchannels the images were constructed from originals using an image processing pipeline as described in Section 2.3. The resulting images show more elongated, directional and interconnected channels that form under limited nutrient supply (50%) in contrast to short and disorganized channels at the center of the fully nutritional macrocolony.

**Figure 1:**
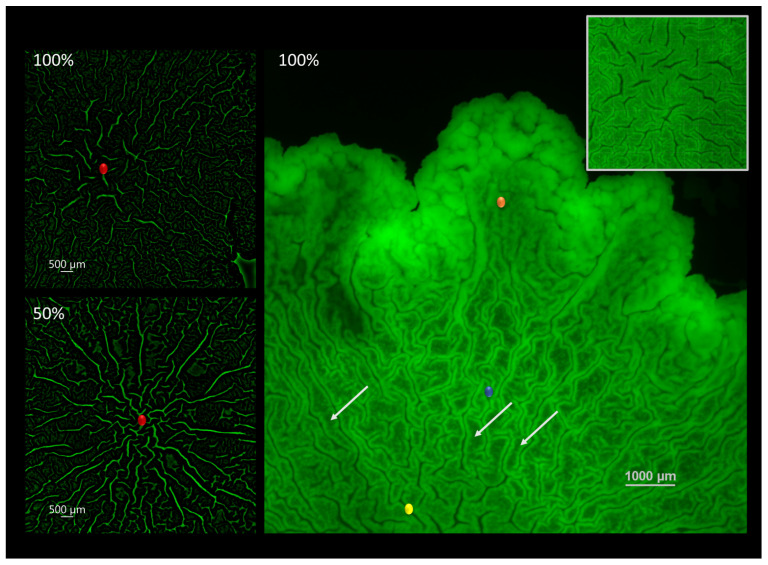
Channel connectivity and organization at the biofilm-agar interface. (**left**) Computerized rendering of a fully nutritional (**top**) and 50% (**bottom**) nutrient-depleted colony core of *B. subtilis* biofilm at the biofilm-agar interface. Center of the colony core is marked in red. (**right**) Contact surface of a fully nutritional *B. subtilis* biofilm. Closeup of the central zone is shown in the top right corner. Several light arrows point to examples of filament-like structures. Scale bar 1000 μm.

Right image shows the different organizational patterns at the biofilm-agar interface of a *B. subtilis* macrocolony grown at full nutrient supply. The biofilm is organized in filament-like folds characterized by a low-intensity middle area flanked by high intensity walls (Figure 1). These structures, to which we refer to as “filaments” in the remainder of this paper are reminiscent of the familiar large-scale channels or “wrinkles” that appear on the biofilm surface—characterized by a weak GFP signal in channel interior and flanked by high-intensity borders or “walls”. Additionally, the surface appears to be zoned—the innermost zone (yellow) at the center is characterized by thicker filaments which are less dense compared to the surrounding zone (blue) which is characterized by thin and tightly packed filaments of a similar structure. Finally, the outermost or leading zone (orange) shows a disperse region which is in the process of organizing into filaments.

### 3.2. Microsphere Dispersal in a Growing Colony

Mature *B. subtilis* colony type biofilms were grown from suspensions that consisted of bacterial cells in a mixture with fluorescent-labeled microspheres as described in Section 2.7. During maturation, microspheres that are embedded in the growing biofilm are displaced according to the forces that are at play during biofilm growth and expansion. The character of these forces can be inferred from the positioning of microspheres as they are seen in mature macrocolonies.

**Figure 2:**
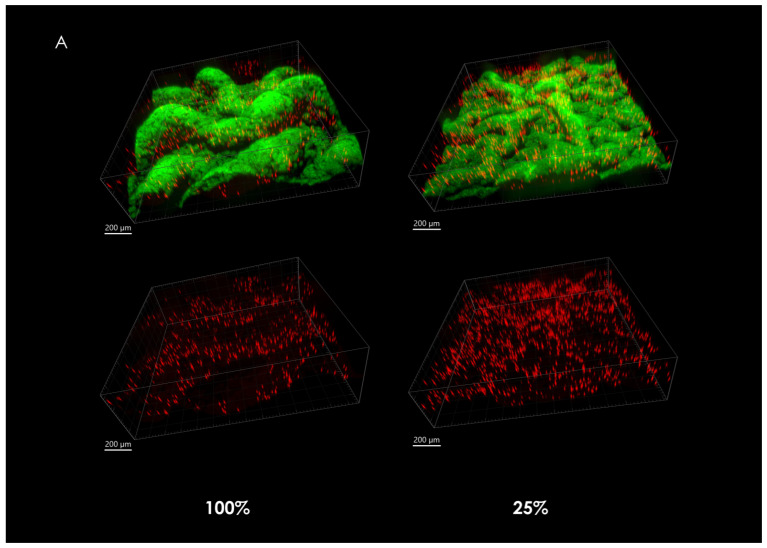

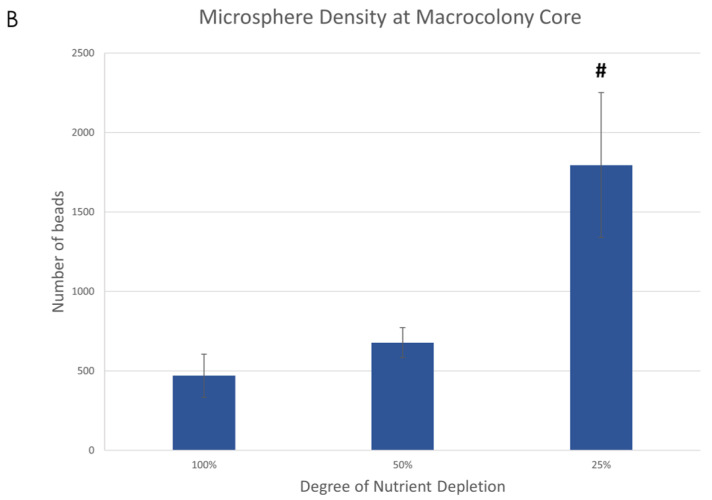
Fluorescent microspheres in mature *B. subtilis* macrocolonies. (**A**) Core of a mature *B. subtilis* colony type biofilm (green) grown in a mixture with red microspheres. (**left** column, top) 100% nutrient supply, (**right** column, top) 25% nutrient supply. Bottom row shows isolated microspheres, corresponding to the top row images. Scale bars 200 μm. (**B**) Number of microspheres found at macrocolonies’ core at 100%, 50% and 25% nutrient supply. The results are representative of eight experiments. # indicates *p* < 0.01.

When observing the overall microsphere density at the center of the macrocolonies upon maturity, it is significantly larger in severely nutrient-depleted macrocolonies (25% nutrient supply) when compared to their 50% and 100% counterparts (Figure 2A). Indeed, in a 1.33 mm^2^ area, macrocolonies with one-fourth of standard nutrient supply contained 382% of the number of microspheres found in the same center area of fully-nutritional macrocolonies (Figure 2B).

### 3.3. Spatial Distribution of Microspheres

**Figure 3:**
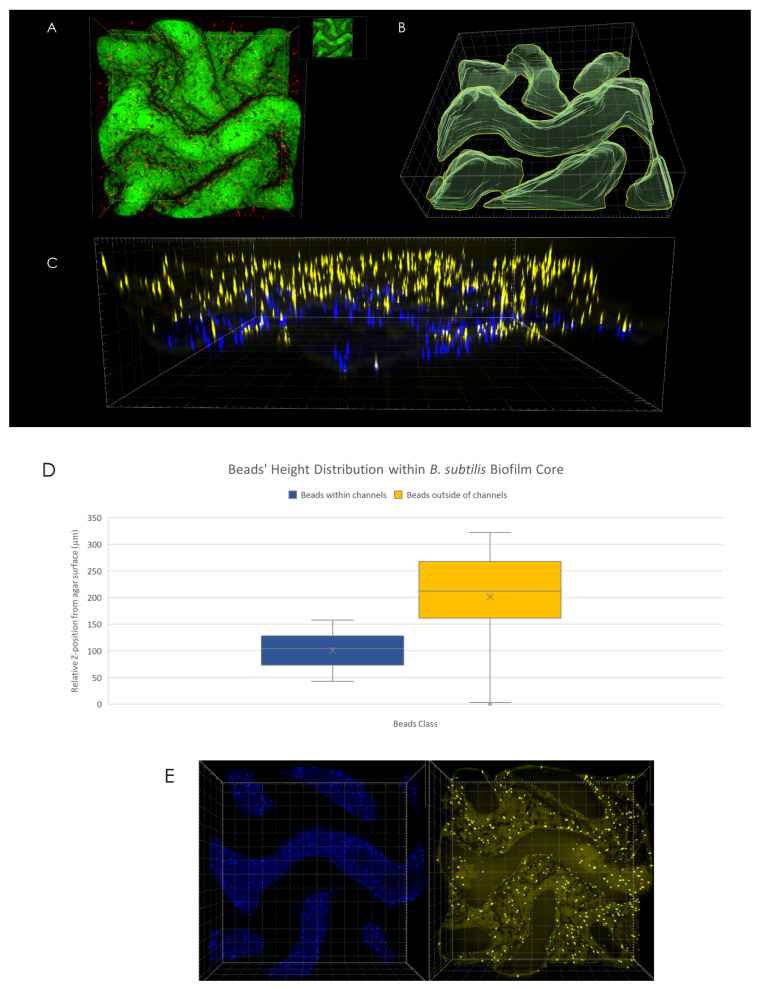
3D microsphere distribution. (**A**) Top view of macrocolony center. (**B**) Channel interior surface render as visualized in Imaris software. (**C**) Side view of microspheres located within (blue) and outside of (yellow) channels’ interior. (**D**) Microspheres positional distribution in Z-axis. (**E**) Top view of microspheres within (**left**) and outside of (**right**) channels’ interior.

An analysis of microsphere position with respect to channel interior (determined via volumetric rendering of channels’ interior as can be seen in Figure 3B) reveals differences in spatial distribution—while no discernible change in microsphere density was observed in X-Y plane (Figure 3E), microspheres located within the interior of channels were found to be located at the bottom of the channels, closer to the agar surface (Figure 3D). For example, in the case of the macrocolony shown in Figure 3A, the difference in mean height between microspheres located within channels’ interior (Figure 3C, blue) was 100 µm compared to the equivalent for microspheres located outside of the channels (Figure 3C, yellow).

### 3.4. Expansion Patterns at the Biofilm-Agar Interface

Microsphere droplets, prepared as described in Section 2.7, were placed onto the surface of the growth media, in the path of expanding macrocolonies. This allows for direct observation of forces that take place at the biofilm-agar interface—as the colonies expand and organize into their characteristic topographical patterns, the microsphere droplets are subject to observable deformation.

**Figure 4:**
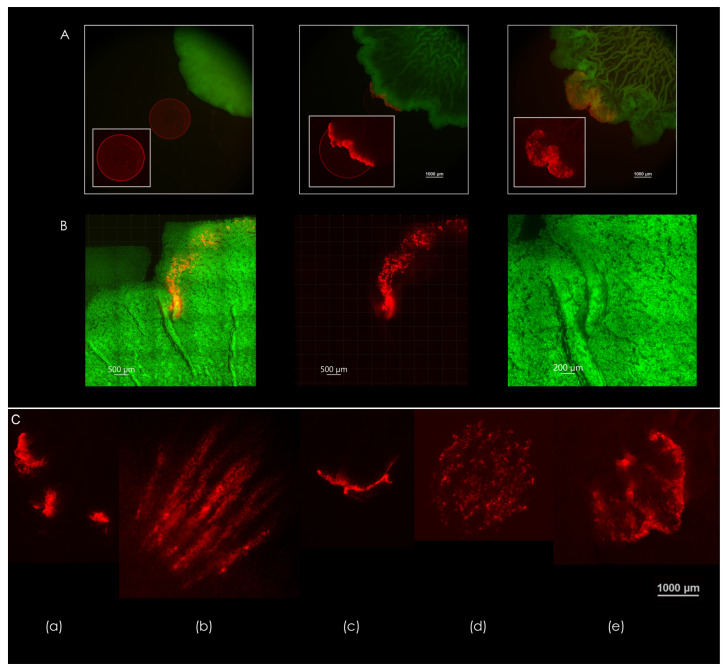
Expansion patterns at the biofilm-agar interface. (**A**) Forces generated by the biofilm as reflected in the deformation of a droplet consisting of fluorescent microspheres (2.5 µL). Days 1-3 are shown, left to right. Scale bars 1000 μm. (**B**) Pathway of microspheres in a droplet caught in a channel formed by a 50% nutrient-depleted macrocolony. Scale bars 500 μm. Rightmost image is a close up of the formed channels—scale bar 200 μm. (**C**) Different modes of deformation found at the contact surface—(**a**) segmented, (**b**) filamentous, (**c**) unidirectional, (**d**) multidirectional, (**e**) turbulent. The images in (**A**,**C**) are representative of eight experiments. Scale bar 1000 μm.

Figure 4A demonstrates the deformation of a microsphere droplet that was seeded in the path of a growing *B. subtilis* macrocolony over a period of three days (left-to-right). Encounter with the macrocolony occurred between days 1 and 2—an undisrupted droplet can be seen in the leftmost image, which was taken on day 1. Middle image shows the deformation of the droplet at the leading edge of the macrocolony. As the colony expands further, the droplet deformation progresses according to the expansion patterns that occur at the leading edge of the macrocolony—this leading edge is in effect the outermost zone, where the colony begins to organize into filaments.

Figure 4B shows the effect of a single channel on the movement of microspheres within the biofilm—note the colocalization of microspheres within channel interior. In contrast, microspheres that did not encounter channel formation move in all directions along with the leading edge of the macrocolony. As the macrocolony matures, various patterns of expansion occur at the contact surface—a few examples of droplet deformations can be seen in Figure 4C.

### 3.5. Macrocolony Impression upon Growth Medium

**Figure 5:**
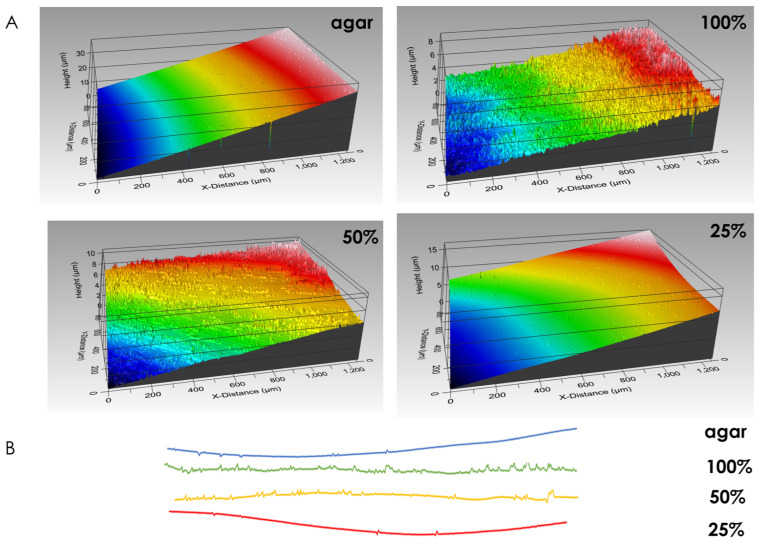
Biofilm footprint upon solid agar medium. (**A**) Surface rendering of growth media after removal of macrocolonies. Control surface on which no bacteria were seeded is shown in top-left image. (**B**) Line roughness profiles corresponding to surfaces in (**A**). The results are representative of six experiments.

Macrocolony footprint upon solid agar medium reveals the differences in modes of adhesion. Fully-nutritional macrocolonies leave behind stronger footprints than their nutrient-depleted counterparts. In fact, at 25% nutrient supply, surface roughness approximates that of a control surface, on which no macrocolony was seeded (Figure 5). Consequently, the forces of adhesion which govern macrocolony attachment to its host surface can be inferred to be weaker in nutrient-depleted macrocolonies. This corresponds to our previous findings that nutrient-depleted macrocolonies increase their expansion rates upon the solid agar medium as faster expansion is facilitated in part by a weaker adhesion to the host surface.

## 4. Discussion

The topographical features of biofilms have been associated in the literature with various functional characteristics. Surface topography was used to estimate such features of biofilms as hydrophobicity [13], wettability, susceptibility to erosion and more. In this paper we explore surface topography at the biofilm-agar interface, where it determines in part the expansion patterns and the modes of adhesion between the macrocolony and growth medium. We present findings of changes that occur at the adhering surface under nutrient depleted growth conditions and demonstrate how specific topographical features of *B. subtilis* biofilms facilitate directional forces within the macrocolonies. Briefly, a network of developed channels at macrocolony core facilitate both an outward-driven force that is directed from core to periphery and a downward-driven force that drives movement of microspheres located within channel interior towards the agar surface. Through the use of fluorescent microspheres we demonstrate three additional types of forces—(a) forces present at the leading edge of the macrocolonies as they mature, (b) forces that localize microspheres within channel interior and (c) forces that occur at the biofilm-agar interface as the macrocolony expands and organizes its morphology during maturation. Finally, we link the topography and expansion patterns to different modes of adhesion of macrocolonies to their growth media and suggest that contact surface topography can be used as an indicator of adhesion.

### 4.1. Biofilm Topography at the Contact Surface Varies both Spatially and under Different Growth Conditions

It has been established in the literature that *B. subtilis* biofilm structures are non-uniform communities of bacterial cells governed by complex gene regulation patterns. Vlamakis et al. [8] demonstrated that different types of bacterial cells (motile, sporulating and matrix-producing) localize at different regions within the biofilm, while Chai et al. [26] showed that specific matrix-relevant genes are expressed in only a subset of cells within the colony type biofilm. The differences in cell differentiation at distinct regions of the mature colonies are detected not only in the agar plane (colony core vs. periphery) but also in the vertical plane—for example, Marlow et al. [27] demonstrated the preferential localization of exoprotease-producing cells closer to the biofilm-air rather than to the biofilm-agar interface. The different cellular and matrix composition at the biofilm-air vs. biofilm-agar interfaces necessitates a bidirectional approach to surface topography—specifically, when analyzing the attachment of biofilms to the agar surface, it is the surface topography at the biofilm-agar interface that is relevant to discussion of adhesion properties. Indeed, in this paper we show that the biofilm-agar interface in a single colony type biofilm exhibits a non-uniform topography which allows for different “zones of adhesion” at the contact surface. The expansion and growth of *B. subtilis* macrocolonies occurs via a continuous maturation process that begins with a disorganized clumping of bacteria (outermost zone), followed by its organization into thin and tightly-packed filament-like dense structures (intermediate zone), which as the colony matures widen and become less tightly packed (innermost zone). As these filaments widen their interior, less GFP signal is observed within each such filament. The overall effect of these morphological changes results in an overall decrease in GFP signal emitted by the biofilm core—when compared to the biofilm periphery, it could explain the previously observed more significant GFP signal intensity reduction at the biofilm core when compared to colony periphery over the maturation period [28]. Thus, this finding is an example of how spatiotemporal topographical changes at the adhering surface of the biofilm may play a role in overall macrocolony morphology and its phenotype.

### 4.2. Microsphere Localization and Dispersal in Growing Macrocolonies

Several past studies focused on determining the types and directionality of forces that take place within the complex architecture of mature *B. subtilis* biofilms. For example, Wilking et al. [29] suggested that the abovementioned network of channels facilitates liquid transport throughout the macrocolonies with evaporative flux acting as the driving force. A complementary force that allows the macrocolonies to uptake nutrients from the growth substrate was also suggested by Gingichashvili et al. [12], while a similar channel-driven nutrient uptake and distribution effect was previously reported in *E. coli* biofilms [30]. Analysis of macrocolonies that were co-cultured and grown in a mixture with fluorescent microspheres reveals two additional forces present in *B. subtilis* macrocolonies. Firstly, as can be seen in Figure 2, microsphere density at the core of nutrient-depleted macrocolonies is significantly greater than the equivalent in fully-nutritional macrocolonies. When comparing the biofilm surface in both cases, it can be seen that in the case of fully-nutritional macrocolonies, the channels that develop at its core (as opposed to macro-channels that connect biofilm core to periphery) are more developed than those on the surface of the nutrient-depleted macrocolony. Consequently, it could be the case that these channels drive the movement of molecules from core to periphery and while it is unclear whether this process occurs primarily in the channel interior or in the regions between the channels, it does suggest an overall enhanced outward force in fully-nutritional macrocolonies.

Moreover, analysis of the spatial distribution of microspheres reveals a preferential positioning of microspheres near the channel bed, rather than an even distribution throughout the channel interior. This suggests a downward-driven force, which could further facilitate expulsion of molecules from the biofilm. It has been previously shown that molecules can be “taken up” from the growth medium by *B. subtilis* biofilms. Here, we demonstrate a complementary force which drives microspheres located within channel interior downward towards the agar surface.

### 4.3. Diversity of Expansion Patterns at the Contact Surface

It has been previously shown that biofilm adhesion to its growth substrate differs according to surface type—this is particularly of interest in the field of dentistry, where a number of studies indicated that such properties as surface roughness, chemical composition and free energy affect biofilm formation in the oral cavity on natural and artificial surfaces [31]. The resulting topography, assembled via the forces that take place at the adhering surface during colony maturation, by extension, is an indicator for the type of adhesion that takes place. In this paper, we show that the forces present at the contact surface of the biofilm can be visualized by the deformation of microsphere droplets placed in the path of biofilm growth. Figure 4C displays a handful of different modes of deformation that were observed at the leading edge of a growing *B. subtilis* macrocolony. The diversity of these expansion patterns may help shed some light on the manner in which *B. subtilis* biofilms organize into their distinctive and highly adaptive morphologies, not only under different environmental conditions but also within each macrocolony—where both spatially and temporally-variant topographical changes can be observed during colony expansion and maturation.

### 4.4. Contact Surface Topography as an Indication of Adhesion Profile

The spatially varying contact surface morphology hints at a likewise non-uniform adhesion “profile”. Indeed, it may provide a prediction model for the adhesion forces present—as the innermost zone of the macrocolony becomes less filament-dense, the attachment to the agar loosens in that area. At the same time, the intermediate region where filaments are tightly packed provides the strongest attachment while the leading zone is again more loosely attached at the leading edge of the biofilm. Under limited nutrient supply, the topographical changes that occur at the contact surface may drive the accelerated expansion of the macrocolonies—their wider and less tightly packed filaments might help explain the enhanced spreading ability of such colony type biofilms [12] as they are likely to result in looser adhesion at the contact surface, which is further supported by profilometry data results.

Finally, the adhesion strength of biofilms to their host surface is considered to be a substantial determinant of virulence and predictor of successful biofilm eradication [32]. If adhesion strength can be inferred from the underlying morphology, without the need to physically disrupt biofilms to analyze their adhesion strength as in [14,17], it would consequently be useful in assessing biofilms’ tolerance and potential virulence. Studies that focus specifically on disease-causing biofilms such as those that form on medical devices or on natural surfaces such as teeth and gingiva would benefit from the analysis of the adhering surface and its topography in addition to erosion assays that could help verify and build such a prediction model.
